# E-prop on SpiNNaker 2: Exploring online learning in spiking RNNs on neuromorphic hardware

**DOI:** 10.3389/fnins.2022.1018006

**Published:** 2022-11-28

**Authors:** Amirhossein Rostami, Bernhard Vogginger, Yexin Yan, Christian G. Mayr

**Affiliations:** ^1^Chair of Highly-Parallel VLSI-Systems and Neuro-Microelectronics, Faculty of Electrical and Computer Engineering, Institute of Principles of Electrical and Electronic Engineering, Technische Universität Dresden, Dresden, Germany; ^2^Centre for Tactile Internet (CeTI) with Human-in-the-Loop, Cluster of Excellence, Technische Universität Dresden, Dresden, Germany

**Keywords:** SpiNNaker 2, E-prop, online learning, training at the edge, parallelism, memory footprint, neuromorphic hardware

## Abstract

**Introduction:**

In recent years, the application of deep learning models at the edge has gained attention. Typically, artificial neural networks (ANNs) are trained on graphics processing units (GPUs) and optimized for efficient execution on edge devices. Training ANNs directly at the edge is the next step with many applications such as the adaptation of models to specific situations like changes in environmental settings or optimization for individuals, e.g., optimization for speakers for speech processing. Also, local training can preserve privacy. Over the last few years, many algorithms have been developed to reduce memory footprint and computation.

**Methods:**

A specific challenge to train recurrent neural networks (RNNs) for processing sequential data is the need for the Back Propagation Through Time (BPTT) algorithm to store the network state of all time steps. This limitation is resolved by the biologically-inspired E-prop approach for training Spiking Recurrent Neural Networks (SRNNs). We implement the E-prop algorithm on a prototype of the SpiNNaker 2 neuromorphic system. A parallelization strategy is developed to split and train networks on the ARM cores of SpiNNaker 2 to make efficient use of both memory and compute resources. We trained an SRNN from scratch on SpiNNaker 2 in real-time on the Google Speech Command dataset for keyword spotting.

**Result:**

We achieved an accuracy of 91.12% while requiring only 680 KB of memory for training the network with 25 K weights. Compared to other spiking neural networks with equal or better accuracy, our work is significantly more memory-efficient.

**Discussion:**

In addition, we performed a memory and time profiling of the E-prop algorithm. This is used on the one hand to discuss whether E-prop or BPTT is better suited for training a model at the edge and on the other hand to explore architecture modifications to SpiNNaker 2 to speed up online learning. Finally, energy estimations predict that the SRNN can be trained on SpiNNaker2 with 12 times less energy than using a NVIDIA V100 GPU.

## 1. Introduction

Nowadays AI applications at the edge are gaining interest. In some real world applications, it is better to process data at the edge. Specifically, there are three main categories where this is important. First, these devices can be used everywhere, especially the places where communication infrastructure is not available. Second, when working with highly sensitive data like medical data, privacy is very important. We do not want to send our private data to the cloud for processing. Third, when a machine or software with Artificial Intelligence (AI) is interacting with the real world, like surgery or autonomous driving, latency plays an important part. We do not like to wait while sending the data to the cloud to be computed and get data back later. In addition, devices which depend on cloud computing are at greater risk of cyber-attack and hacking. By omitting the communication between edge and cloud, we can reduce power consumption of devices and increase their lifetimes (Sze et al., 2017; Chen and Ran, 2019; Cai et al., 2020).

We are living in a dynamic world and due to many reasons such as aging, changing seasons, new construction, and pandemic (Bavel et al., 2020), our behavior and environment change continuously. Edge devices such as smartphones, wearable smartwatches, Internet of Things, and autonomous vehicles are used in our daily life. Data privacy for these devices is very important and we do not like to collect user data and send it to cloud for further processing. Some examples include learning user behavior in smartphones or learning modifications in the Internet of Things applications like smart home. One may use federated learning to train and update models on these devices in such a way that the data are local (Li et al., 2020b).

For reasons mentioned in previous paragraphs, in some applications, there is a need to continue training at the edge. But the problem is that training on edge devices is challenging as we have access to limited memory and clock frequency, and power budgets of these devices are limited.

In this paper our focus is mainly on learning at the edge, with limited memory and clock cycles. Specifically, we are interested in online training of sequential data. Recurrent neural networks (RNNs), because of their sequential nature, are widely used in temporal applications like machine translation, speech recognition, image tagging, and so on.

Compared to ANNs, SNNs are more energy efficient and they are neuromorphic hardware friendly because they use spikes (one bit data) to communicate to other neurons (Poon and Zhou, 2011; Tavanaei et al., 2019). In this paper, our focus is on training SRNNs. By using surrogate gradients (Neftci et al., 2019), one can train SRNNs through the Back Propagation Through Time (BPTT) method. The main drawback of BPTT is that, it is not a memory efficient algorithm, because in the backward path it needs information of all previous time steps in the forward path. Also BPTT is not an online learning rule, because when it processes data in the forward path it could not update the weights and it should come back to the first time step. Then there is enough information to update weights. For these two reasons, BPTT in not an edge device-friendly learning rule. One of the algorithms that solves these issues is E-prop (Bellec et al., 2020a), a memory efficient online learning mechanism for SRNN. In E-prop, memory storage is *O*(*N*^2^) where *N* is the number of recurrent neurons, it is an online and spike-based algorithm. Similar to other online learning algorithms that use approximation to compute gradients, E-prop loses some accuracy compared to BPTT, too.

Our purpose here is to implement E-prop on the SpiNNaker 2 hardware. SpiNNaker 2 is an ARM-based multi-core neuromorphic system and it is specially designed for simulating spiking neural networks. The final SpiNNaker 2 chip will consist of 152 processing elements (PEs) containing an ARM cortex-M4F core with 128 KB SRAM memory. By a dedicated on-chip packet router and chip-to-chip links, one can connect thousands of chips in a hexagonal grid to form a large SpiNNaker machine with millions of cores (Furber et al., 2014; Mayr et al., 2019). What makes SpiNNaker a “neuromorphic” system is (1) the scalable event-based communication (Navaridas et al., 2015), and (2) the massively parallel real-time operation where the cores are only loosely synchronized by a regular timer event to start the computation of neuron updates in the software.

In this work, we implement the training process of an SRNN from scratch by using the E-prop algorithm in a SpiNNaker 2 prototype. First, we use a single processing element (PE) for evaluating a small SRNN. The network contains only 20 recurrent neurons due to memory and computing limitations. Then we expand our work and implement a larger network (120 recurrent neurons) on 12 PEs. The network and the E-prop training is parallelized by dividing the synapses among PEs and using spikes as the main communication mechanism. We verify our implementation result with a TensorFlow implementation of the E-prop algorithm for the Google Speech Commands dataset. Further, we perform a clock profiling analysis to find bottlenecks and learn how we could improve the speed by integrating a hardware accelerator in future work. This is complemented by fitting models to predict the timing (number of clock cycles) of the main algorithm functions to see how the E-prop scales up. In addition to this, we compare the memory consumption of E-prop and BPTT in detail and analyze when either of the two is more memory efficient while achieving the same accuracy.

Our main contributions for short, are as follows:

- We implement the E-prop algorithm on a neuromorphic system.
- We successfully train a SRNN by using E-prop from scratch on a neuromorphic system.
- We provide a parallel implementation of E-prop on a multi-core neuromorphic system to achieve real-time operation.
- We verify that the E-prop learning rule works with a mini batch size of one.
- We perform a clock profiling analysis to find the bottleneck for future hardware accelerators.
- We conduct a memory and clock cycle analysis of E-prop in comparison to BPTT.

The remainder of the article is structured as follows. Section 2 introduces the considered SRNN, the E-prop algorithm, the SpiNNaker 2 hardware system, and describes in detail the E-prop implementation on SpiNNaker 2. In Section 3 we compare the SpiNNaker 2 E-prop results to a TensorFlow reference and provide further analysis on the memory and computing cost. Finally, in Section 4 we provide a thorough discussion on related work for memory-efficient online learning (in theory and on neuromorphic hardware) and compare our results to state-of-the-art SNNs for the Google Speech Commands dataset.

## 2. Materials and methods

### 2.1. Network model and training

#### 2.1.1. Network architecture

**Figure 1:**
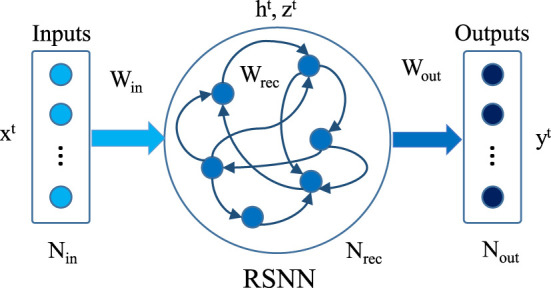
Schematic of recurrent spiking neural network. The middle layer consists of spiking recurrent neurons.

We used a two-layer SRNN architecture as our model. The architecture is shown in Figure 1. Here *N*_*in*_ denotes input neurons, *N*_*rec*_ represent recurrent spiking neurons, and *N*_*out*_ is output neurons. The input neurons are connected to the recurrent neurons with weights *W*^*in*^. *W*^*rec*^ shows the recurrent weight matrix and *W*^*out*^ represents the weights between recurrent neurons and output neurons. All weight matrices are dense. *x*^*t*^ is the input vector at time step *t*; note that the inputs $x_{j}^{t}$ have floating-point values, not spikes. *h*^*t*^ is the hidden state of the spiking neurons (e.g., representing the membrane potential) and *z*^*t*^ is a binary vector representing the spikes in time step *t*. The output neurons just sum up the received synaptic events and are represented by the scalar output vector *y*^*t*^.

#### 2.1.2. Spiking neuron models

In this paper we consider two kinds of spiking neuron models in the recurrent layer: the leaky-integrate-and-fire (LIF) neuron and the Adaptive LIF (ALIF) model. The dynamics of the LIF neuron model is defined by the following equations:


(1) $$\begin{array}{l} v_{j}^{t+1}=\alpha v_{j}^{t}+\underset{i\neq j}{\sum }W_{ji}^{rec}z_{i}^{t}+\underset{i}{\sum }W_{ji}^{in}x_{i}^{t+1}-z_{j}^{t}v_{th} \end{array}$$

(2) $$\begin{array}{l} z_{j}^{t}=H\left(v_{j}^{t}-v_{th}\right) \end{array}$$

Where $v_{j}^{t}$ is the membrane potential, $\alpha =e^{-\delta t/\tau_{m}}$ is the decay factor with δ*t* being the time step duration, τ_*m*_ the membrane time constant, and *v*_*th*_ the spike threshold. *H* expresses the Heaviside step function and is used to detect a spike. Hence, $z_{j}^{t}$ is a binary variable where its value is 1 if neuron *j* spikes in time step *t*, and 0 otherwise. When a neuron sends a spike, its membrane voltage is decreased by *v*_*th*_ (last term in Equation 1).

As networks of LIF neurons have poor capabilities for processing longer-term patterns, Bellec et al. (2018) have introduced the ALIF neuron model. They added an adaptive threshold to the LIF neuron model to add long short-term memory capabilities to SRNNs. The dynamics of the ALIF neuron model are defined by Equation (1) for the membrane potential and the following equations:


(3) $$\begin{array}{ll} A_{j}^{t} & =v_{th}+\beta a_{j}^{t} \end{array}$$

(4) $$\begin{array}{ll} z_{j}^{t} & =H\left(v_{j}^{t}-A_{j}^{t}\right) \end{array}$$

(5) $$\begin{array}{ll} a_{j}^{t+1} & =\rho a_{j}^{t}+z_{j}^{t} \end{array}$$

Here, $A_{j}^{t}$ is the adaptive threshold potential, with the adaptive component $a_{j}^{t}$ and a scaling constant β. $\rho =e^{-\delta t/\tau_{a}}$ is a decay factor of the adaptive component with τ_*a*_ being the adaptation time constant. The adaptive threshold is increased by β for each spike and decays back exponentially to the baseline threshold. When LIF and ALIF neurons are combined in the recurrent layer in Figure 1, the model is called Long short-term memory Spiking Neural Network (LSNN) (Bellec et al., 2018).

We evaluated LIF, ALIF, and LSNN in our simulation and we found that when adding LIF to ALIF neurons, the memory and computation increases but test accuracy is not considerably affected. So we decided to use only ALIF neurons in our model. We used a discrete-time model of ALIF with time step δ*t* = 10*ms*. In our model we defined the ALIF neuron parameters as: τ_*m*_ = 5, β = 0.184, τ_*a*_ = 150 and *v*_*th*_ = 0.01.

#### 2.1.3. Surrogate gradient

One problem of spiking recurrent neural networks (SNNs) is that the spike function (Equation 2) at the occurrence of spikes, is not differentiable. Hence, unlike the conventional neurons in RNNs, spiking neurons are not appropriate for standard gradient-based optimization. To overcome this limitation, we use the surrogate gradient method (Neftci et al., 2019) with a multivariate Gaussian function (Yin et al., 2021) as an approximation to the derivative of the spike function. Concretely, we approximate the partial derivative $\partial z_{j}^{t}/\partial v_{j}^{t}$ by the multivariate Gaussian function $\psi \left(v_{j}^{t}\right)$:


(6) $$\begin{array}{l} \psi \left(v_{j}^{t}\right)=\left(1+h\right)N\left(v_{j}^{t}|0,\sigma^{2}\right)-hN\left(v_{j}^{t}|\sigma ,{\left(s\sigma \right)}^{2}\right)-hN\left(v_{j}^{t}|-\sigma ,{\left(s\sigma \right)}^{2}\right) \end{array}$$

where $N\left(v_{j}^{t}|\mu ,\sigma^{2}\right)$ is the Gaussian distribution with mean μ and standard deviation σ. Here σ is 0.5 and hyperparameters *h* and *s* are 0.15 and 6, respectively.

#### 2.1.4. E-prop algorithm

There are two main methods used to train RNN in machine learning: Back Propagation Through Time (BPTT) (Werbos, 1990) and Real-Time Recurrent Learning (RTRL) (Williams and Zipser, 1989).

For BPTT, during the forward path one needs to store the hidden state variable of all time steps. Then, in the backward path for computing the gradients of loss function with respect to network parameters (weights), one starts from the last time step, uses stored data from the forward path and goes back to the previous time step, and uses that information until reaching the first time step. Finally the gradients can be computed and the weights can be updated.

There are two problems with this method. First, the memory usage increases with the number of time steps, and second, it is not an online learning mechanism. This means that when the algorithm computes the last time step, it can not immediately compute the gradients and update weights. Instead, BPTT needs to iterate back to the first time step; only then all the necessary information is available to compute the gradients. In other words, a system with BPTT is a non-causal system and it is not an appropriate fit for online real-time systems.

However, the RTRL algorithm solves these two problems. In the forward path, the RTRL algorithm carries additional information from the current to the next time step. At the last time step, all the information to compute the gradients and update the weights is available. On the other hand, still two new drawbacks come with RTRL: It requires almost one order of magnitude more memory than BPTT and also the computation time is two orders of magnitude larger than BPTT. For more information, please look at the research of Marschall et al. (2020).

**Figure 2:**
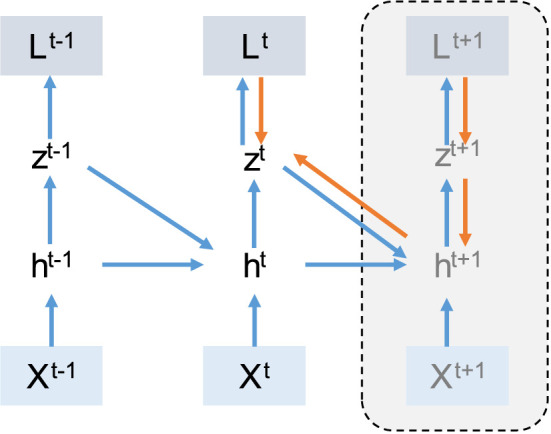
Computation graph for E-prop: In E-prop, unlike BPTT, for computing the gradient in the current time step, we do not need the information from next time step (dashed line). In this case in time step *t* we have enough information to compute gradients. In picture the blue arrows show the forward path and the orange arrows show the backward path.

In recent years many algorithms such as KF-RTRL (Mujika et al., 2018), UORO (Tallec and Ollivier, 2018), KeRNL (Roth et al., 2018), RFLO (Murray, 2019), SnAp (Menick et al., 2020), E-prop (Bellec et al., 2020a; Zenke and Neftci, 2021), SuperSpike (Zenke and Ganguli, 2018), DECOLLE (Kaiser et al., 2020), and OSTL (Bohnstingl et al., 2022) were developed to simplify the RTRL method. These algorithms actually tried to make some approximation on RTRL. In RTRL, the memory complexity is *O*(*N*^3^) and computation is *O*(*N*^4^), where *N* is the number of recurrent neurons. These algorithms reduce the memory complexity to *O*(*N*^2^) and computation to *O*(*N*^2^) or *O*(*N*^3^) at the cost of losing accuracy. One of these algorithms is E-prop (Bellec et al., 2020a). In E-prop (Bellec et al., 2020a), information that is not accessible in the current time step is ignored. In Figure 2 this information is shown by the dashed line. E-prop tries to train SRNN without the knowledge of future time steps. The E-prop algorithm is an online learning method and compared to BPTT is more memory efficient. This claim is discussed in more detail in Section 4.1.

In E-prop, the gradient of loss function with respect to weights is computed as:


(7) $$\begin{array}{l} \nabla_{W}L=\overset{T}{\underset{t=1}{\sum }}{\text{λ}}_{j}^{t}{ē}_{ji}^{t} \end{array}$$

The first term is called the learning signal and is computed as:


(8) $$\begin{array}{l} {\text{λ}}_{j}^{t}=\frac{\partial L_{j}^{t}}{\partial h_{j}^{t}}=\underset{k}{\sum }B_{jk}\left(y_{k}^{t}-y_{*,k}^{t}\right) \end{array}$$

where *j* denotes the number of recurrent neurons and *k* denotes the number of output classes, * denotes ground truth labels and *B*_*jk*_ are neuron-specific weights. They can be equal to the output weights (symmetric E-prop), or selected randomly (random E-prop) or evolve through a simple local plasticity rule (adaptive E-prop). In our model we use symmetric E-prop because we could achieve better accuracy. The second term in Equation (7) is called eligibility trace and is computed as:


(9) $$\begin{array}{l} e_{ji}^{t}=\psi_{j}^{t}\left({\bar{z}}_{j}^{t-1}-\beta \epsilon_{ji,a}^{t}\right) \end{array}$$

where $\psi_{j}^{t}$ is the surrogate gradient of membrane potential function in Equation (5) and the bar sign means a low pass filter and can be computed as:


(10) $$\begin{array}{l} {\bar{z}}_{j}^{t}=k{\bar{z}}_{j}^{t-1}+z_{j}^{t} \end{array}$$

where *k* is the decay factor. In Equation 9, $\epsilon_{ji,a}^{t}$ is the eligibility vectors for adaptive threshold:


(11) $$\begin{array}{l} \epsilon_{ji,a}^{t}=\psi_{j}^{t}\epsilon_{ji,v}^{t}+\left(\rho -\psi_{j}^{t}\beta \right)\epsilon_{ji,a}^{t-1} \end{array}$$

where $\epsilon_{ji,v}^{t}$ is eligibility vectors for the membrane potential and its value for input and recurrent neurons can be computed as follows:


(12) $$\begin{array}{l} \epsilon_{ji,v}^{t,in}={\bar{x}}_{j}^{t} \end{array}$$

(13) $$\begin{array}{l} \epsilon_{ji,v}^{t,rec}={\bar{z}}_{j}^{t-1} \end{array}$$

actually eligibility vectors for the membrane potential are matrices where rows are repeated.

#### 2.1.5. Network output and loss functions

The output neurons could be computed as:


(14) $$\begin{array}{l} y_{k}^{t}=Cy_{k}^{t-1}+\underset{j}{\sum }W_{kj}^{out}z_{j}^{t}+b_{k}^{out} \end{array}$$

where subscript *k* denotes the number of output neurons (here number of categories), constant C defines the leakage and $b_{k}^{out}$ shows the bias term. For the classification task we chose cross-entropy loss function and it is computed as:


(15) $$\begin{array}{l} L=-\underset{t,k}{\sum }\pi_{k}^{*,t}\pi_{k}^{t} \end{array}$$

where * denotes the ground truth label and $\pi_{k}^{t}$ is:


(16) $$\begin{array}{l} \begin{array}{l} \pi_{k}^{t}=softmax(y_{k}^{t})=\frac{y_{k}^{t}}{\sum_{k}exp(y_{k}^{t})} \end{array} \end{array}$$

For computing the gradient of the output weights, there is no need to apply the E-prop learning rule, instead we can compute it as follows Bellec et al. (2020b):


(17) $$\begin{array}{l} \text{Δ}W_{kj}^{out}=\underset{t}{\sum }\left(\pi_{k}^{t}-\pi_{k}^{*,t}\right){\bar{z}}_{j}^{t} \end{array}$$

### 2.2. Dataset and preprocessing

We applied E-prop to train a RSNN for key word spotting using the 12-category Google Speech Commands (GSC-12) dataset (Warden, 2018). The primary goal of the GSC dataset is to provide a way to build and test small models that are suitable for edge devices. The model detects what word is spoken, from a set of ten target words, with as few false positives as possible from background noise or unrelated speech.

Before the audio signals move into the model, they should be preprocessed. For preprocessing, we use a similar approach as Zimmer et al. (2019). We remove extra information from the audio signal, the model becomes simpler and also it is more robust to noise. A method that is widely used in literature is Mel-Frequency Campestral Coefficients (MFCCs). In this method, log Mel filters and their first and second-order derivatives are extracted from raw audio signals.

For the FFT, we used a window size of 30 ms and a hop length of 10 ms. Then, the log of 40 Mel filter coefficients was extracted with LibROSA (McFee et al., 2015) using a Mel scale between 20 and 4,000 Hz, as this frequency band contains the most information. Finally, the spectrograms corresponding to each derivative order are re-scaled. After preprocess, the one second input audio is converted to a 100 time steps signal. The preprocess task has been done offline.

### 2.3. Implementation of E-prop on the SpiNNaker 2 FPGA prototype

#### 2.3.1. SpiNNaker 2 FPGA prototype

SpiNNaker 2 is the second generation of SpiNNaker chip, a digital neuromorphic system developed by Technische Universität Dresden and University of Manchester (Mayr et al., 2019).

**Figure 3:**
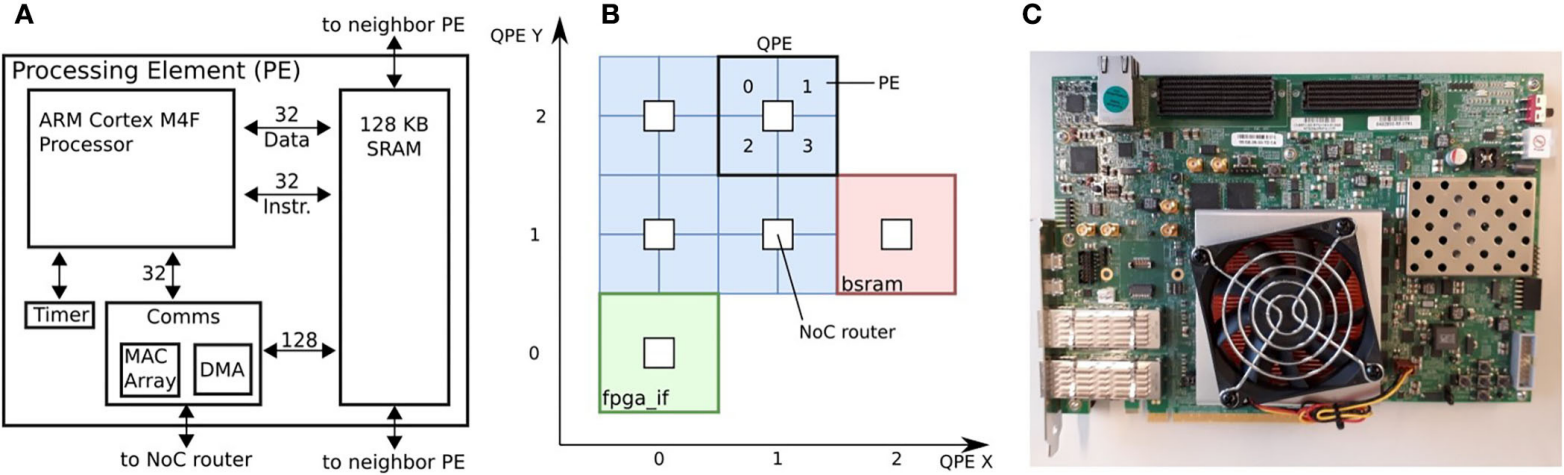
SpiNNaker 2 FPGA prototype: **(A)** Architecture of SpiNNaker 2 processing element, **(B)** Down-scaled SpiNNaker 2 architecture with 16 processing elements on 4 QPEs, host interface (fpga_if) and block SRAM (bsram), **(C)** Board photo (Xilinx Virtex UltraScale+ FPGA VCU118 evaluation board).

The final SpiNNaker 2 chip will contain 152 ARM Cortex-M4F cores or processing elements (PEs) which support single-precision floating-point operation. The simplified block diagram of a PE is shown in Figure 3A. Each PE contains 128 KB SRAM, from which typically 32 KB is used for instruction and 96 KB for data memory. Four PEs form a Quad PE (QPE) and they have direct access to the memory of other PEs in the same QPE with only a few clock cycles latency. The PEs can communicate *via* the Network-on-Chip (NoC) and each QPE contains one router. The communication unit (Comms) uses NoC packets to send spikes to other PEs. Furthermore, it supports a direct memory access (DMA) module to transfer a block of data from one PE to another PE (it could be in another QPE). Each PE further contains an array of 64 multiply-accumulate (MAC) units to speed up 8-bit or 16-bit integer operations for matrix multiplication or 2D convolution operations (not used in this work). See Höppner et al. (2021) for further details on the chip and PE architecture and Yan et al. (2019) and Yan et al. (2021) for applications of specific hardware features.

In our work, we use a SpiNNaker 2 FPGA prototype (Huang et al., 2022), the architecture is shown in Figure 3B and the board picture in Figure 3C. In Figure 3B, the fpga_if is the Ethernet based interface between host PC and PEs. The prototype contains four QPEs (hence 16 PEs) and a block SRAM (bsram) of 64 MB. The latter has been foreseen as a substitute for the missing DRAM in this setup, but is not used in this work. We use the Comms unit to send spikes to all PEs *via* the Network-on-Chip (NoC), the timer interrupt to call main function every 10 ms (one time step), the timer register for clock profiling, and the DMA to transfer data between PEs at the last time step, and the fpga_if to debug code and read or write from/to internal PEs memory. On the FPGA, the PEs are clocked at 65 MHz.

The main advantage of SpiNNaker 2 over SpiNNaker 1 (Painkras et al., 2013; Furber et al., 2014) for training neural networks is that SpiNNaker 2 supports single-precision floating-point arithmetic operations in the hardware, while SpiNNaker 1 supports only fixed-point operations. In addition, there is more flexibility in the communication, such as more flexible chip-to-chip packets and a high-bandwidth network-on-chip for core-to-core data transmission. Finally, SpiNNaker 2 has a higher performance per chip with 152 instead of 18 core that can run faster up to 300 MHz while being more energy-efficient.

#### 2.3.2. Implementation of E-prop on a single processing element

As discussed in Section 2.2, after the preprocessing step, every one second audio signal is converted to 100 time steps frequency data. Correspondingly for real-time implementation, every time step can be processed in 10ms time intervals. In other words, every 10 ms a time step is processed.

First, we implemented E-prop on a single PE with the C programming language and using only internal memory of the PE. We reserved 24 KB for stack and debugging, so 72 KB are free for variables usage. The large variables and their memory consumption is shown in Table 1. The storage for ALIF neurons, hyperparameters and output neurons is not shown in this table, because they consume only a small portion of memory. The input matrix is the preprocessed audio data, cf. Section 2.2.

**Table 1:** Large E-prop variables and memory consumption, memory is calculated for time step=100, *N*_*in*_ = 80, *N*_*rec*_ = 20 and *N*_*out*_ = 12.

	**Variable**	**FP32**	**KByte**
Input	*X*[*N*^*in*^][*time*_*step*]	8,000	31.25
E-prop	$\epsilon_{a}^{in}\left[N^{in}\right]\left[N^{rec}\right]$	1,600	6.25
	$\epsilon_{v}^{in}\left[N^{in}\right]\left[N^{rec}\right]$	1,600	6.25
	*e*^*in*^[*N*^*in*^][*N*^*rec*^]	1,600	6.25
	ē^*in*^[*N*^*in*^][*N*^*rec*^]	1,600	6.25
	$\epsilon_{a}^{rec}\left[N^{rec}\right]\left[N^{rec}\right]$	400	1.56
	$\epsilon_{v}^{rec}\left[N^{rec}\right]\left[N^{rec}\right]$	400	1.56
	*e*^*rec*^[*N*^*rec*^][*N*^*rec*^]	400	1.56
	ē^*rec*^[*N*^*rec*^][*N*^*rec*^]	400	1.56
	λ[*N*^*rec*^]	20	0.08
Weights and gradients	*W*^*in*^[*N*^*in*^][*N*^*rec*^]	1,600	6.25
	*W*^*rec*^[*N*^*rec*^][*N*^*rec*^]	400	1.56
	*W*^*out*^[*N*^*rec*^][*N*^*out*^]	240	0.94
	Δ*W*^*in*^[*N*^*in*^][*N*^*rec*^]	1,600	6.25
	Δ*W*^*rec*^[*N*^*rec*^][*N*^*rec*^]	400	1.56
	Δ*W*^*out*^[*N*^*rec*^][*N*^*out*^]	240	0.94
ADAM optimization	*m*^*in*^[*N*^*in*^][*N*^*rec*^]	1,600	6.25
	*v*^*in*^[*N*^*in*^][*N*^*rec*^]	1,600	6.25
	*m*^*rec*^[*N*^*rec*^][*N*^*rec*^]	400	1.56
	*v*^*rec*^[*N*^*rec*^][*N*^*rec*^]	400	1.56
	*m*^*out*^[*N*^*rec*^][*N*^*out*^]	240	0.94
	*v*^*out*^[*N*^*rec*^][*N*^*out*^]	240	0.94
Sum		24,980	97.57

FP32 denotes how many 32-bit memory locations it occupies.

The E-prop algorithm needs storage for the input membrane potential eligibility vector $\epsilon_{v}^{in}$ (Equation 12), the input adaptive threshold eligibility vector $\epsilon_{a}^{in}$ (Equation 11), the recurrent membrane potential eligibility vector $\epsilon_{v}^{rec}$ (Equation 13), and the recurrent adaptive threshold eligibility vector $\epsilon_{a}^{rec}$ (Equation 11). Also it needs storage for the input eligibility trace *e*^*in*^ and recurrent eligibility trace *e*^*rec*^ for the current time step and to apply the decay (the low pass filter) to the previous time step (ē^*in*^ and ē^*rec*^) (Equation 9). We need storage for input, recurrent, and output weights (*W*^*in*^, *W*^*rec*^ and *W*^*out*^), and gradients of these weights (Δ*W*^*in*^, Δ*W*^*rec*^ and Δ*W*^*out*^). ADAM optimization needs storage for first and second moment vectors (*m* and *v*) for all weights (Kingma and Ba, 2017).

The procedure for implementing the E-prop learning rule on SpiNNaker 2 is as follows. Before starting training, the dataset is preprocessed once using the MFCC algorithm. The dataset size is about 4 GB and it is stored in the host PC connected to the SpiNNaker 2 prototype. The host PC reads the dataset and writes it to the internal memory of SpiNNaker by using the Ethernet connection and NoC packets. As shown in Table 1, each input data for one sample requires 31.25 KB memory. To reduce the required memory, only 10 time steps of input data are moved to the PE SRAM; once the data has been processed, new data is transferred. To decrease the waiting time for the input data, we used a ping-pong buffer. The host PC writes 5 time steps to the ping buffer and 5 time steps to the pong buffer and triggers SpiNNaker 2 to start operation. After the data in each buffer is used, the host writes the new input data.

After reading one time step from the input buffer, the SpiNNaker 2 core computes the part of the membrane voltage that needs input data (Equation 1). Before updating the neuron model, it computes the parts of Equations (10), (12)–(14) that need previous time step information.

Then, according to the ALIF neuron model (Equations 1–5), the membrane potentials of all neurons are calculated. If the neuron membrane potential value is above the adaptive threshold, the neurons send spikes. For the one PE implementation there are only internal spikes.

Then, the E-prop parameters such as eligibility vector for input $\epsilon_{a}^{in}$, eligibility vector for recurrent layer $\epsilon_{a}^{rec}$, eligibility traces for input *e*^*in*^, and eligibility traces for recurrent layer *e*^*rec*^ of current time step are calculated according to Equations (9–13). After computing eligibility traces in the current time step, the low-pass filter in Equation (10) is applied to them, because we need ${ē}_{ji}^{t}$ for gradients computation (Equation 7). After that, the parts of Equations (1), (10), (12), and (14) that need the current time step information are computed.

The processes described above are repeated for all time steps. After processing all time steps, the average of the output is computed, and sparse softmax cross entropy is applied to the output and the target output (Equation 16) to compute the loss function (Equation 15). Then it computes the learning signal (Equation 8), and the gradients of input, recurrent (Equation 7), and output weights (Equation 17). Finally, by using ADAM optimization, the weights are updated. This is the process for one iteration (one sample of the dataset) and it is repeated for all iterations to generate the loss and accuracy result for one epoch. The pseudo code for the E-prop implementation is shown in Algorithm 1. A summary of functions and their descriptions are provided in Table 2.

**Algorithm 1:**
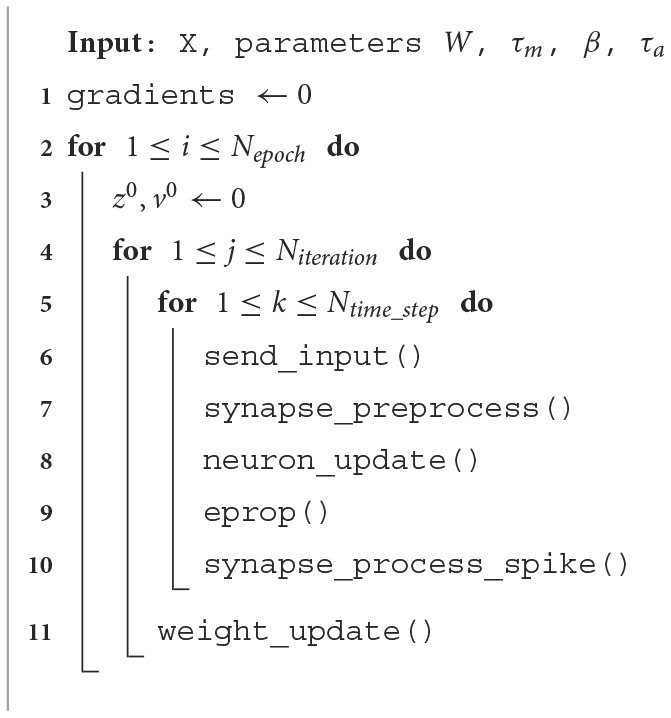
One core E-prop implementation.

**Table 2:** E-prop algorithm functions and what they do.

**Function**	**Description**
send_inputs	Read input data from ping-pong buffer
synapse_preprocess	Compute previous time step part of equations
neuron_update	Update ALIF neurons state
E-prop	Compute eligibility traces and gradients of weight w.r.t loss function
synapse_process_spike	Compute current time step part of equations
weight_update	Compute gradients, apply ADAM optimization and update weights

We used TensorFlow 1.14 with an NVIDIA Tesla V100 SXM2 32 GB GPU for simulation. For the validation of the C code in the SpiNNaker 2 prototype, we used the same initial weights, which were randomly generated in TensorFlow. We also used a single-precision floating-point format (float32) for variables in TensorFlow and C. We compared the results of each step in C and TensorFlow to debug and validate our implementation.

#### 2.3.3. Parallelization approaches

**Figure 4:**
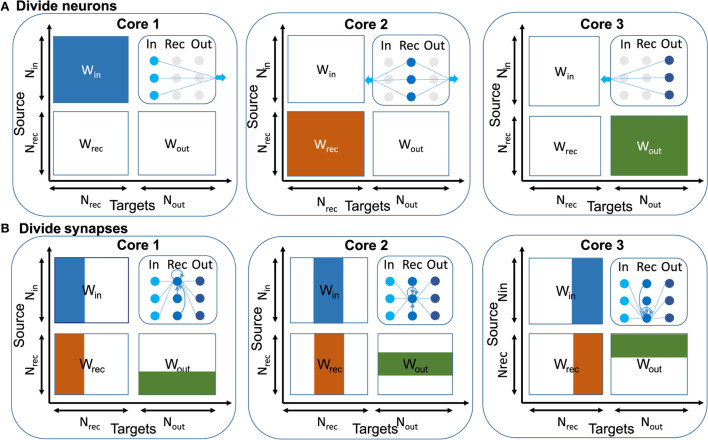
Illustration of parallelization approaches based on connection matrices of the RSNN. **(A)** Divide neurons: In this approach each core is responsible for a specific neuron type and all the synapses. Here some cores do more computation than the other cores. The blue arrows in the top row show that there are large data movements between cores. **(B)** Divide synapses: In this approach post-synaptic connections are divided between cores and each core is responsible for all pre-synaptic connections and part of post-synaptic connections. Here we can divide computation symmetrically between cores.

In machine learning, mini-batch size is usually between 32 and 100, so common machine learning frameworks such as TensorFlow and PyTorch can execute input data independently in parallel on multiple CPU or GPU cores to accelerate the training (Abadi et al., 2016; Li et al., 2020a). But in online learning the mini-batch size is one. This means we only have access to one input, so this method is not applicable here. Our main aim in this section is to implement a relatively large model at edge scale (120 recurrent neurons) on the multi-core SpiNNaker 2 system. The algorithm should work in real-time. If one uses only a single core, the available compute clock cycles per 10 ms time step are only sufficient to process networks with up to 76 recurrent neurons, so that the 120 neuron model could not run in real-time. We talk about memory usage in more detail in the Supplementary material. To solve this problem, one should distribute the computation in two or more cores. There are two main approaches to scale up the design and parallelize the online learning rule: neuron-based and synapse-based. The block diagram of these two approaches is shown in Figure 4.

In the first approach one can distribute neurons among multiple cores and each core is responsible for computing the parameters of specific neurons. For example, core 1 deals with input neurons and computes the gradient of the loss function with respect to the input weights and updates input weights, but core 1 needs the learning signals for computing gradients and should wait for it. Core 1 sends the result of input current to core 2 which is responsible for the recurrent neurons. This core uses this information, computes the current and membrane potential, and generates spikes, also it is responsible for computing recurrent gradients and updating recurrent weights. Just like core 1, core 2 also needs learning signals for computing gradients. Finally, core 3 needs spike information to compute the error and generate output. Learning signals are also computed in core 3. Core 1 and 2 need this information to compute gradients. In this method core 2 executes more computation than core 1, and core 1 executes more computation than core 3, because in typical neural networks the number of recurrent neurons is larger than the number of input neurons and the number of input neurons is larger than the number of output neurons.

In the second approach one can distribute synapses among multiple cores and each core is responsible for computing a specific part of the synapse computation. The main difference between this method and the first method is that, in the previous method, one neuron and its synaptic connections are implemented in one core but in this method synaptic connections for one neuron are divided between several cores. In other words, all computations are divided equally between cores, and each core is responsible for a part of the computation of a neuron, not all of them.

For the E-prop learning rule, it turns out that most of the calculation related to computing gradients and updating weights can be executed locally, so that at each time step only spikes need to be sent between cores. Instead, the transmission of error signals is performed only at the last time step (time step number 100). This enables a seamless scaling to many cores while maintaining a real-time operation during the 100 steps.

Except for input neurons and some parameters related to output neurons, other parameters have a dimension equal to the recurrent neurons. In E-prop, the computations related to the recurrent dimension are independent and one can divide parameters from this dimension among several cores. In this method, one core is the main core and it is responsible for collecting the error result of other cores in last time step, calculating the final error, and sending it to other cores. So other cores can compute parts of the gradients and update parts of the weights.

In the neuron-distribution method (first method) data movement and extra memory usage at the end of the last time step, is *O*(*N*_*rec*_). One needs extra memory to store data coming from other cores. Each core is programmed with a different code. Implementing a larger network needs to compute how many cores one requires for input, recurrent, and output neurons. So also one should consider how to implement each neuron again. In the second level of parallelization one could benefit from the second approach and implement a mixture of neuron-synapse-distributions. Also in this method each core is idle for a long time before data coming from other cores is available.

In the synapse-distribution method (second method), data movement and extra memory usage at the end of the last time step is *O*(*N*_*out*_). It means this method is more memory efficient. All cores run programs with the same code. So code maintenance in the synapse-distribution method is easier. Also implementing a larger network and using more cores is easier, because computation is distributed equally between cores. In our work, we implement the second approach. A similar approach was used in Liu et al. (2018) for non-spiking deep neural networks.

As E-prop is the RTRL approximation, we do not have weight transposition in the gradient computation path. This means that almost all of the computations related to weights and gradients can be done locally. The synapse-based approach can be applied to the E-prop and other similar learning rule algorithms, for scaling the model in size. In some learning rules such as BPTT, if one uses a synapse-based approach, there would be a large data movement for transferring gradients.

#### 2.3.4. Implementation of E-prop on multiple processing elements

In this section, we describe the difference between the one-PE and the multi-PE implementation in the SpiNNaker 2 prototype. In the multi-PE implementation, the host PC sends input data to all PEs. When there is a spike, it is sent to all neurons in other PEs. For this purpose we use the SpiNNaker Comms unit in multicast (MC) mode. This unit is specially designed to send spikes to other cores. We used a synchronization flag to make sure all spikes from other PEs are taken into account before continuing processing.

After all time steps are processed, the output should be calculated. Each PE calculated a fraction of output (Equation 14). To compute the final output, total loss (Equation 15), and accuracy, this data is transmitted to PE0. PE0 uses the SpiNNaker 2 DMA controller for this task. All PEs wait in this step for a synchronization flag. They wait for the output error, i.e., the difference between estimated output and ground truth output. They need it for calculating the learning signal [Equation (8)] and to calculate the gradients of weights (Equation 7). PE0 computes the output error and then transmits it to other PEs. Also PE0 sends a synchronization flag to other PEs, so they know that it is time to resume computation.

If one wants to update the weights in each time step instead of only in the last time step, this process should be repeated in every time step and, as it will be shown in Section 3.2.2, the number of MAC operations would increase approximately two-fold. For a summary of the multi-PE algorithm, look at Algorithm 2. The *sync*() function is used for the synchronization between PEs.

**Algorithm 2:**
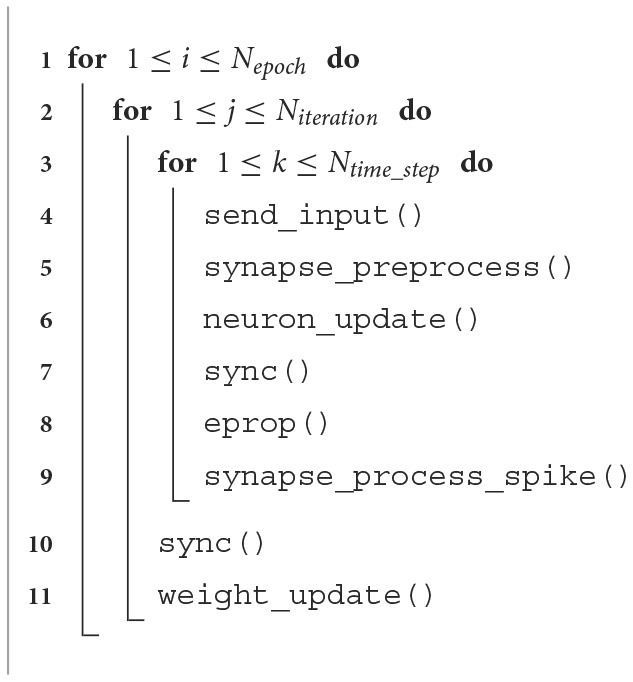
Multi-core E-prop implementation.

## 3. Results

In this part, we show the results of the E-prop implementation on the SpiNNaker 2 prototype. First, in Section 3.1, we searched for optimum network hyperparameters. Then, in Section 3.2, we compared the simulation result in TensorFlow to the SpiNNaker 2 prototype implementation in terms of accuracy, and we performed a clock profiling and analyzed how much time was spent in each function. In Section 4.1, we compared the memory footprint of E-prop and BPTT. In Supplementary material, we developed and fit simple models to predict the number of clock cycles for every function in the algorithm to see how they scale up when increasing the network size. There, we performed some experiments to compute the number of clock cycles with respect to network parameters. We found that if we know the clock speed of processor (150 MHz to 300 MHz for SpiNNaker 2 chip and 65 MHz for the prototype), we can compute how many clock cycles are available in the 10 ms interval and by comparing the result, one could check if the algorithm could run online.

### 3.1. Network study

In this section, we explored the optimum values for network hyperparameters. First we searched for the appropriate number of input and recurrent neurons, and then we changed the network parameters to find the optimal network for better accuracy of the validation dataset. To prevent an overfitting on the training dataset, we applied L2 regularization. In principle, it is also possible to apply firing rate regularization to limit spike generation frequency. However, we found that this parameter increases the network complexity and also reduces the final accuracy. Hence, we did not use firing rate regularization.

For the optimization process, we tried Stochastic Gradient Descent (SGD), Momentum, RMSProp, and ADAM optimization. We would rather choose SGD, as it does not require extra memory matrices for storing the mean and/or the second momentum of gradients. However, SGD and Momentum could not optimize the network at all and RMSprop achieves 1% less accuracy compared to the ADAM optimizer. So we decided to use the ADAM optimizer.

In MFCC, one can change the number of mel frequencies by changing the delta order. As it is shown in Table 3, this parameter affects the number of input neurons and the dataset size. *N*_*in*_ is the number of input neurons.

**Table 3:** Effect of delta order in input and database size.

**Delta order**	***N*_*in*_**	**Train dataset**
0	40 x 1	1.9 GByte
1	40 x 2	3.9 GByte
2	40 x 3	5.9 GByte

Accordingly, it is desirable to use a smaller delta order for the purpose of decreasing the model complexity and reducing the dataset size. In our simulation we also consider this hyper parameter. The simulation result for different model parameters is shown in Table 4. *N*_*adt*_ is the number of ALIF neurons. In the following simulation, L2 regularization is 10e-5.

**Table 4:** Comparison of model parameters on accuracy.

**Network params**		**Trainable params**	**Train acc. (%)**	**Best valid acc. (%)**	**Test acc. (%)**
***N*_*in*_**	***N*_*rec*_**				
40x1	40	4K	85.78	83.22	82.34
40x1	120	21K	90.68	86.84	86.76
40x2	40	5K	91.81	88.69	88.51
40x2	80	14K	94.65	89.73	90.67
40x2	88	16K	94.84	90.02	90.48
40x2	120	25K	95.90	90.76	91.07
40x3	40	7K	93.13	88.86	88.7
40x3	80	17K	93.89	90	90.71
40x3	120	30K	97.2	91.1	91.61
40x3	160	47K	97.96	91.56	91.68
40x3	360	180K	99.01	91.85	92.4

We decided to use *N*_*in*_ = 40x2 and *N*_*adt*_ = 120. Furthermore, we found that by reducing the L2 regularization to 0.05e-5, we obtained a better result. With these parameters we achieved 91.2 ± 0.16% accuracy on the test set in less than 22 epochs on average in 10 runs.

### 3.2. SpiNNaker 2 implementation

In this section, we compared the epoch-accuracy diagram in test and validation sets between our implementation in SpiNNaker 2 and TensorFlow software simulations. Also we did a clock profiling in hardware and performed a detailed memory comparison between E-prop and BPTT. In the Supplementary material, we performed a hardware architecture exploration for implementing E-prop on SpiNNaker 2. More specifically, we considered the use of the SRAM from neighbor PEs to increase the memory per PE, and derived models to predict the number of clock cycles when scaling up the network size.

#### 3.2.1. Comparison of SpiNNaker 2 implementation and TensorFlow

During training E-prop in TensorFlow, the mini batch size was 100 and we initialized the learning rate to 0.01. But for online training the batch size is 1, and also the learning rate should be selected again. We tried different learning rates such as 0.01, 0.0003, and 0.001 for more than 4 epochs on the SpiNNaker 2 prototype and we observed that training with the 0.001 learning rate converges faster. So we performed the experiment with the 0.001 learning rate.

**Figure 5:**
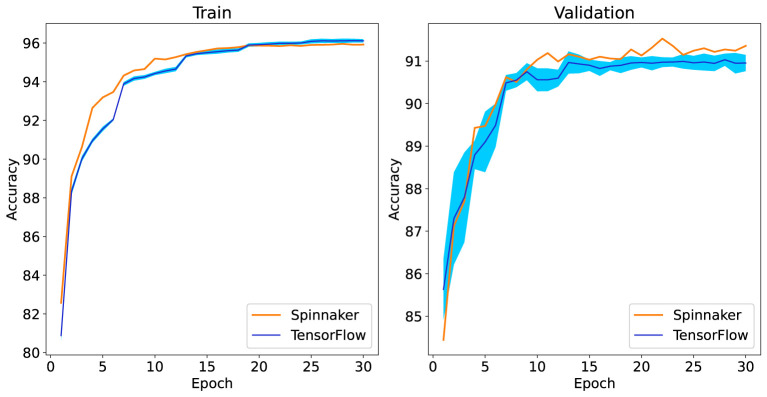
Comparison of train and validation set accuracy result for TensorFlow simulation and SpiNNaker 2 implementation. The TensorFlow results are shown for 10 runs, the dark blue line shows the average and the light blue cloud shows the standard deviation of 10 runs. The SpiNNaker 2 result is for one run.

For training on the SpiNNaker 2 prototype, one epoch of training for the GSC dataset (60,000 iterations) takes about 20 h. So 30 epochs take more than 24 days. Also it takes 4 h to achieve accuracy of the validation and test sets. In GPU with a batch size of 100, the whole process (30-epochs) takes about 2 h. With a batch size of one, the whole process takes 17 days (Estimated after running some iterations). In Figure 5, the train and validation set accuracy for the TensorFlow simulation and the SpiNNaker 2 implementation is shown. The TensorFlow results are shown for 10 runs, while the SpiNNaker 2 result is given only for a single run due to the long training time. In TensorFlow, the average test accuracy is 91.2%. In the SpiNNaker 2 prototype, the best valid accuracy is in epoch 22 and the test accuracy for this epoch is 91.12%. It seems that the training convergence in SpiNNaker 2 is a little faster (especially in the first epochs) than TensorFlow, due to using a smaller mini batch size.

We note that the very long training time on SpiNNaker 2 is due to the implementation being optimized for online learning (batch size one) with real-time input (each 1 s audio sample is processed by a SRNN in 1 s wall clock time). To increase the training speed in SpiNNaker 2, one can use a larger mini batch size and always 12 PEs are responsible for one batch. Then for implementing a 100 mini batch, 1200 PEs are needed.

#### 3.2.2. Clock profiling

**Figure 6:**
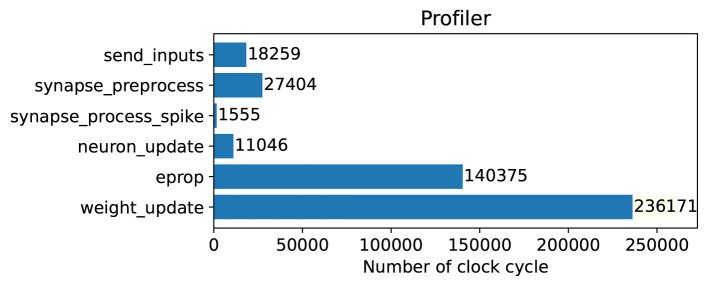
Clock profiling in SpiNNaker 2 FPGA prototype for one PE that runs in parallel with 11 other PEs simultaneously to execute the whole model. The weight_update runs every 100 time steps and all other functions run in every time step.

In this section, we performed clock profiling to find out where there is a bottleneck in terms of clock usage and what should be improved on the hardware side in the future. We measured the number of clock cycles needed by each function. The function descriptions were shown in Table 2. We used the internal timer register counter to measure and calculate the clock cycles per function. In Figure 6, the clock cycle profiling for the algorithm run in SpiNNaker 2 is shown. Every 100 time steps, the *weight*_*update*() function is run once to update weights and other functions run every time step. So one PE needs about 200 K clock cycles to process the data from one time step and it should be done within the 10 ms time interval.

As it is clear, if the weights were updated in every time step, the number of clock cycles would be doubled roughly. As it is shown in this figure, about 70% of clock cycles per time step is spent in the E-prop function to calculate gradients of loss function with respect to weights. Also we analyzed the E-prop function further. Moreover, 80% of the E-prop function is used for matrix multiplication and 20% for matrix addition. Matrix multiplication is used to compute eligibility traces of the E-prop algorithm. To improve hardware performance, a wise choice would be to use matrix-by-matrix multiplication hardware accelerator that support floating-point data type, to perform this task faster. It is worth mentioning that in SpiNNaker 2 there is a 16 × 4 MAC array per PE, which, however, only supports fixed-point data types and hence is not appropriate in our case.

#### 3.2.3. Power and energy consumption

In Table 5, power and energy results for GPU and SpiNNaker 2 are shown. For a batch size of 100, the GPU needs 1 h and 58 min and consumes 657kJ energy (see Supplementary Section S3 for details). In our experiments, we used an FPGA prototype of SpiNNaker 2 which consumes significantly more power than the final SpiNNaker 2 chip does. Hence, it would not be meaningful to provide FPGA power results. Instead, we provide a coarse approximation based on measurements of a prototype chip in Höppner et al. (2021) which reported an efficiency for the CoreMark benchmark of 21.59 μW/MHz at 100 MHz and 0.5 V for one SpiNNaker 2 core. We estimate that each of the 12 cores used in the E-prop implementation consumes the same power as for the CoreMark where the PE is active all the time, yielding a total power consumption of 26 mW. To run the training in real-time for 30 epochs with 70,375 1-s GSC audio files each, it takes 586 h and 27 min. Hence, we estimate an energy of 54.7 kJ for training the model on SpiNNaker 2 which is about 12 times more efficient than the GPU. In both cases, the power of the host CPU is not considered. We remark that this is only a rough approximation and that the actual energy consumption on the SpiNNaker 2 chip may deviate.

**Table 5:** Power and energy consumption: For NVIDIA Tesla V100 SXM2 32 GB GPU the batch size is 100 and the average power usage during training is calculated.

**Measurement**	**NVIDIA**	**SpiNNaker 2 (estimation)**
Batch size	100	1
Power [W]	91.3	0.026
Time [h:m]	1:58	586:27
Energy [kJ]	646.4	54.7

For SpiNNaker 2, we assume a real-time operation with a 100 MHz clock frequency. See text for details on the estimation.

## 4. Discussion

### 4.1. Memory analysis of E-prop and BPTT

In this section, we are interested in quantifying the memory benefit of using E-prop instead of BPTT for our SpiNNaker 2 implementation. For BPTT we can divide the gradients computation in two paths, forward and backward. In the forward path, the state variable in every time step is computed and it is stored. In the backward path the algorithms start from last time step and come back to first time step to compute gradients. In every time step it uses state variables in the forward path and also gradients of next time steps. For online learning algorithms like E-prop, there is only one forward path. In the forward path, the state variable and gradients information are computed at the same time step.

In the literature, the memory usage of BPTT is mentioned as *O*(*NT*), where *N* is the number of neurons. It seems that only the memory utilization for storing the hidden state variable of the forward path is considered and the memory usage for computing gradients in the backward path is not considered. But in online learning algorithms the overall memory consumption is calculated. So for a fair comparison between BPTT and the others, one should consider both forward and backward paths. In this case the correct memory consumption for BPTT is *O*(*N*^2^+*NT*).

For a fair memory comparison between E-prop and BPTT, we considered an optimized implementation of both algorithms and assumed that all variables have a single-precision floating-point format. Then we counted the variables that are needed for all time steps. The result is shown in Table 6.

**Table 6:** Memory comparison between E-prop and BPTT.

**Algorithm**	**Memory consumption**
E-prop	2*N*_*out*_+*N*_*in*_+6*N*_*rec*_+7(*N*_*in*_×*N*_*rec*_)+7(*N*_*rec*_×*N*_*rec*_)+4(*N*_*rec*_×*N*_*out*_)
BPTT	*E*−*prop*+*N*_*time*_*steps*_×(*N*_*out*_+*N*_*in*_+3 × *N*_*rec*_)

**Figure 7:**
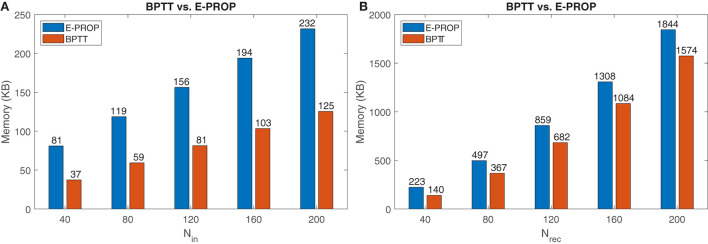
Memory comparison between E-prop and BPTT: **(A)** By changing the number of recurrent neurons. **(B)** By changing the number of time steps and comparing the memory for BPTT (256 neurons) and E-prop (360 neurons).

By using information in Table 6, we plotted the diagrams in Figure 7. For a model with 80 input, 20 recurrent, 12 output neurons, and 100 time steps, E-prop consumes 56 KB and BPTT consumes 119 KB of internal memory. So E-prop can be implemented in one PE's memory but BPTT needs two PE's memory. For 120 recurrent neurons, BPTT needs about 180 KB more memory than E-prop. As it is clear from Table 6, the BPTT memory usage increases linearly by increasing the number of time steps but E-prop does not depend on the time steps. The number of time steps limits applications and model complexity of BPTT at edge devices.

According to Tables 4, 7, E-prop with 360 neurons could achieve almost the same accuracy as BPTT with 256 neurons. Now the question is, whether E-prop with more neurons needs more memory or BPTT with less neurons to achieve the same accuracy.

**Table 7:** Comparison of SNN and RNN for Google Speech Commands dataset with respect to memory and accuracy.

**Paper**	**Architecture, training**	**Hiddenunits**	**Modelsize**	**Memory**	**Accuracy(%)**
This work	ALIF, E-prop	120	25 K	680 KB	91.2
Yin et al. (2021)	ALIF, BPTT	256	167 K	2.7 MB	92.1
Pellegrini et al. (2021)	NLIF, 3 layer Conv2D	-	130 K	5.9 MB	94.5
Salaj et al. (2021)	LSNN (SFA), BPTT	2,048	4 M	120 MB	91.2
Zhang et al. (2018)	CNN + DS-CNNx4 + FC, BP	-	38 K	692 KB	94.4
Kusupati et al. (2018)	FastGRNN, BPTT	100	1.4 K	760 KB	92.10
de Andrade et al. (2018)	Conv2Dx2 + Bi-LSTMx2, BPTT	64 x 4	202 K	41.5 MB	95.6

The model size represents the number of trainable parameters. The memory includes all parameters and temporary variables for training the model with 100 time steps at batch size 1.

By using equations in Table 6, it is clear that E-prop with 360 neurons needs 4.3 MB and BPTT with 256 neurons needs 2.7 MB memory. But BPTT needs more memory as the number of time steps increases. To find out the number of time steps where BPTT needs more memory than E-prop, we assume 256 neurons for BPTT and 360 neurons for E-prop and vary the time steps. The result is shown in Figure 7B. When the number of time steps is 600, then both BPTT and E-prop need almost the same memory. But as we increase the number of time steps to more than 600, BPTT needs more memory.

As shown in this section, we cannot generally state that online learning algorithms like E-prop are more memory efficient than BPTT. Instead, we should consider the number of time steps and also the accuracy. For a small number of time steps, BPTT is a better choice as we could achieve a better accuracy by using a smaller network and the same memory, but when the number of time steps increases, E-prop becomes a better choice, because we could implement a larger network with same memory usage.

### 4.2. Related work on memory efficient online learning

In Marschall et al. (2020) a comprehensive overview on algorithms that approximate the RTRL influence matrix (derivative of hidden state variables with respect to network parameters) by a lower dimension matrix is provided.The Kronecker-Factored RTRL (KF-RTRL) algorithm (Mujika et al., 2018) benefits from the Kronecker product decomposition to approximate the gradients. The Unbiased Online Recurrent Optimization (UORO) algorithm (Tallec and Ollivier, 2018) made a rough estimate of the influence matrix by the outer product of two vectors. For this purpose, they used a rank-one unbiased approximation to matrix *M*. The Kernel RNN Learning (KeRNL) algorithm (Roth et al., 2018) is inspired by the node perturbation method (Werfel et al., 2003) to approximate matrix *M*. Random-Feedback Online Learning (RFLO; Murray) (Murray, 2019) made two approximations: first they remove the nonlocal term from the gradient, and second they use random feedback weights to carry errors back to the network. Sparse n-Approximations (SnAp) (Menick et al., 2020) claimed that sparsely connecting a large number of neurons is better than densely connecting a smaller number in terms of calculation. To achieve this goal, they enforced sparsity on matrix *M*.

In addition to these, over the last few years online learning algorithms for neuromorphic systems were developed as well. These biologically plausible algorithms are used for training spiking neural networks (SNNs). E-prop (Bellec et al., 2020a) followed a similar approach like RFLO. They start with BPTT, removed the terms related to future time steps and used local information to compute gradients. Zenke and Neftci (2021), motivated by approaches in RNN, provide a framework for neuromorphic online learning algorithms. They divide the Jacobian matrix in implicit and explicit parts and showed that the explicit term is a dense matrix while the implicit term is a sparse matrix. So they removed the explicit term. SuperSpike (Zenke and Ganguli, 2018) used a nonlinear Hebbian three-factor rule to update synaptic weight. Deep Continuous Local Learning (DECOLLE) (Kaiser et al., 2020), which is based on SuperSpike, utilizes layer-wise local readouts to compute gradients locally. Online spatio-temporal learning (OSTL) (Bohnstingl et al., 2022) divided the gradient flows into three parts, spatial, temporal and mixed trace, and they ignore the last part. They proclaimed that deep SRNN could be trained online.

Also, recently some related work has been done on neuromorphic hardware. In Frenkel and Indiveri (2022) they introduced the ReckOn chip with simplified E-prop. For this purpose they applied space and time locality and sparsity in weight updates to E-prop. In Perrett et al. (2022), they implemented E-prop on the first generation of SpiNNaker. The authors used three cores, one for input neurons, one for hidden neurons (LIF and ALIF), and one for readout neurons. They trained an SNN network without recurrent connections for wave-form matching and temporal credit assignment tasks. In comparison, we provide a parallel implementation on 12 PEs and use a recurrent hidden layer.

### 4.3. Overview of SRNN algorithms on GSC dataset

In this section, we compare our result with other spiking algorithms that used a GSC dataset. Yin et al. (2021) used GSC, with ALIF neurons. They used a multivariate Gaussian surrogate gradient with BPTT to train SRNN. Pellegrini et al. (2021) used non-leaky-integrate-and-fire (NLIF) neurons with a 3-layer convolution neural network (CNN). Salaj et al. (2021) used spike frequency adaptation (SFA) neurons and applied BPTT to train the network.

For a more complete comparison, we also consider a non-spiking algorithm. In Kusupati et al. (2018), FastGRNN is mainly designed to train the neural network in GPU and deploy it in microcontrollers with 32 KB RAM. For this reason it contains three stages: learning low-rank representation, learning sparsity structure, and optimization with fixed-point parameter support. The training epoch in each state is 100 and overall it takes 300 epochs. For offline training, it is fine. Also note that for GSC-12 they used a subset of the dataset (about half of the data) for train and test sets. In de Andrade et al. (2018), a slightly more complex network with a 10-channel Conv2D and filter size of (5 x 1), a 1-channel Conv2D with filter size of (5 x 1) followed by 2 bidirectional LSTM with 64 recurrent neurons and some dense layer is used.

In Table 7, we compare these algorithms. Memory usage is considered for batch size of one and for 100 time steps (1 s) for the whole training process (forward and backward paths). All algorithms mentioned in Table 7 except FastGRNN can be trained in about 20 epochs.

For our work, Salaj et al. (2021) and Yin et al. (2021), we computed the memory usage by using equations in Table 6. For Pellegrini et al. (2021), as a rough estimate, we computed the memory for three convolution layers, multiply it by two (as the backward path needs storage for gradients which have the same dimension as weights) and add storage for three input channels.

In Zhang et al. (2018), first they trained the model by using a 32-bit floating point data format on a GPU for different architectures such as depthwise separable CNN (DS-CNN), CNN, and GRU. Then they quantized the deep neural network (DNN, a standard feed-forward neural network) model and applied inference on the ARM Cortex-M7 microcontroller which occupied about 70 KB memory. For a fair comparison we consider training DS-CNN model with a 32-bit floating point data format. It needs at least 692 KB memory for training; the memory calculation is described in Supplementary Section S2.

For Kusupati et al. (2018), we considered the equations in the paper, in forward and backward paths to estimate the memory usage. For de Andrade et al. (2018), they fed an input of size 80 x 125 to the network, where the number of time steps is 125. We used this information to compute the forward path memory computation and as an approximation multiply it by 2 to consider forward and backward path memory usage. As it is clear, compared to other spiking neural networks, E-prop needs less memory and still can achieve an acceptable accuracy.

### 4.4. Memory efficient online learning

We implemented E-prop, an online memory efficient algorithm for training spiking recurrent neural networks on SpiNNaker 2, a multi-core neuromorphic system. Training 30 epochs with a mini batch size of one takes about 24 days in SpiNNaker 2, if the algorithm works in real-time. In a real-world application, one could pre-train E-prop using a GPU and store network parameters such as weights, learning rates, and optimization variables (mean and second moment in ADAM). These pre-trained parameters could be used as the initial parameters at the edge and one could continue learning on the device. As the algorithm runs real-time, it could learn new data, when it arrives. For instance, the model could learn a new keyword or adapt to a new speaker.

Moreover, we analyzed memory usage and computation of the E-prop algorithm and back propagation through time. With same computation, E-prop consumes less memory, at the cost of losing accuracy. We showed that, if we train a larger network by E-prop, we can achieve almost the same accuracy. But if the number of time steps is 100, E-prop with a larger network needs more memory than BPTT with a smaller network for the same accuracy. If we increase the number of time steps, after 600 time steps E-prop uses less memory. When online behavior of an algorithm is not important for a small number of time steps, BPTT is a better choice and for a larger number of time steps E-prop is a better choice. In addition we discussed parallelization strategies and implemented a 120 spiking recurrent neural network on a multi-PE SpiNNaker 2 prototype. For this purpose we used 12 PEs.

The next step would be to reduce the memory footprint further by using quantization (Acharya et al., 2022) or mixed-precision training (lower precision data format for some variables) (Micikevicius et al., 2018; Kalamkar et al., 2019), low-rank matrix representation (Kusupati et al., 2018) and also using sparsity (Liu et al., 2018).

Deploying online learning in an actual hardware system is not a straightforward task. Implementing an online learning rule is just one part of it. Also the system needs to know when to start learning. As we showed, about 70% of computations are used for computing gradients and updating weights. So it is not energy efficient to always apply learning to new samples. In addition, if we train our network with new data, after some iterations, the network will forget what it learned previously (catastrophic forgetting). So we need other methods to prevent this (Saha et al., 2022).

In conclusion, in this paper we implemented E-prop, a biologically plausible online learning rule, on 12 cores of SpiNNaker 2, a digital multi-core neuromorphic system. We showed that E-prop could be used in hardware for training spiking recurrent neural networks, even with a batch size of one. Moreover, we estimated the power and energy usage in SpiNNaker 2 and compared it with GPU and we showed that although SpiNNaker 2 that works in real-time is 300 times slower than GPU, it is about 12 times more energy efficient. For future work, we plan to extend the model and use larger models on SpiNNaker 2. In the end, it is worthwhile to mention that biologically plausible algorithms need more research to become comparable to traditional ANNs in terms of achieving better accuracy and using less memory at the same time. One possible approach could be to use more complex neuron models with more parameters.

## Data availability statement

Publicly available datasets were analyzed in this study. This data can be found at: https://download.tensorflow.org/data/speech_commands_v0.01.tar.gz.

## Author contributions

AR and BV conceived the study. AR carried out the simulation in TensorFlow, implemented the E-prop algorithm in C/C++, and performed the experiment on the SpiNNaker 2 prototype chip. AR measured the metrics with the aid of BV and YY. CM supervised the findings of this work. All authors discussed the results and contributed to the final manuscript.

## Funding

This work has received funding from the ECSEL Joint Undertaking (JU) under grant agreement No. 876925 (ANDANTE). The JU receives support from the European Union's Horizon 2020 research and innovation programme and France, Belgium, Germany, Netherlands, Portugal, Spain, Switzerland. This work has received funding from the EU Horizon 2020 framework under grant agreement 945539 (Human Brain Project SGA3). This work was partially funded by the German Research Foundation (DFG, Deutsche Forschungsgemeinschaft) as part of Germany's Excellence Strategy-EXC 2050/1-Project ID 390696704-Cluster of Excellence Centre for Tactile Internet with Humanin- the-Loop (CeTI) of Technische Universität Dresden. This work was partially funded by the German Federal Ministry of Education and Research (BMBF) and the free state of Saxony within the ScaDS.AI center of excellence for AI research.

## Conflict of interest

The authors declare that the research was conducted in the absence of any commercial or financial relationships that could be construed as a potential conflict of interest.

## Publisher's note

All claims expressed in this article are solely those of the authors and do not necessarily represent those of their affiliated organizations, or those of the publisher, the editors and the reviewers. Any product that may be evaluated in this article, or claim that may be made by its manufacturer, is not guaranteed or endorsed by the publisher.
