# Supplementary material for: E-prop on SpiNNaker 2: Exploring online learning in spiking RNNs on neuromorphic hardware
Source: Front Neurosci. 2022 Nov 28;16:1018006. doi: 10.3389/fnins.2022.1018006 (PMC9742366; doi:10.3389/fnins.2022.1018006)
Supplement: Supplementary file 1 [file Data_Sheet_1.PDF]

Supplementary Material

1 ARCHITECTURE EXPLORATION

In this section we are interested to see how e-prop scales up when increasing the network size. In SpiNNaker2, one PE can also access other memories in the same QPE. The access to extra memory is slower compared to the internal memory and it takes two or more clock cycles for read or write. We compare the computation and memory result with respect to network parameters for one PE that have access only to its own memory and one PE that have access to extra memory.

1.1 Fitted functions for one PE

We run the program on SpiNNaker2 FPGA prototype for 30 different number of input, recurrent and output neurons and measure the clock cycles for each function. Then we use the data and a non-linear least squares method to fit a function for computing clock cycles with respect to network parameters. Also we used the coefficient of determination (R-squared) to measure how well the function fitted to practical data. The result is shown in Table S1.

Table S1. Fitted functions for clock cycles for one PE

function	Clock	R_squared
send_input	$11.32 N_{rec} N_{in} + 316.18$	0.9982
synapse_preprocess	$145.14 N_{rec} + 13.16 N_{rec} N_{in}$ $+12.43 N_{rec} N_{out} + 837.20$	0.9981
neuron_update	$544.04 N_{rec} + 173.00$	1
eprop	$65.12 N_{rec} N_{in} + 56.81 N_{rec} N_{rec} + 4368.80$	0.9976
weight_update	$103.32 N_{rec} N_{in} + 138.03 N_{rec} N_{out}$ $+62.88 N_{rec} N_{rec} + 9969.50$	0.9969
Total_no_update	$1037.6 N_{rec} + 38.31 N_{in} + 85.90 N_{rec} N_{in}$ $+19.72 N_{rec} N_{out} + 54.31 N_{rec} N_{rec} + 47948.00$	0.9999

1.2 Fitted functions for one PE with two memories

In SpiNNaker2 one PE can access other PE's memory in one Quad PE (QPE), but at least it takes two cycles for a write operation and three cycles for a read operation. As discussed in the paper, the eprop function uses most of the PE clock cycle. So it is reasonable to keep variables related to the eprop function in the main and fast PE's memory and move other variables to second and slower memories.

We changed the number of input, recurrent and output neurons and run the algorithm for 54 different model parameters on the SpiNNaker2 PE and measured the clock cycle for each function of the algorithm. Again, we fitted models to the data, the results are shown in Table S2.

Table S2. Fitted functions for clock cycles for one PE with two memories

function	clock	R_squared
send_input	$16.67N_{rec}N_{in} + 655.02$	0.9966
synapse_preprocess	$13.03N_{rec}N_{in} + 18.19N_{rec}N_{out} + 9.26N_{rec}N_{rec} + 941.58$	0.9959
neuron_update	$540.73N_{rec} + 0.11N_{rec}N_{in} + 0.70N_{rec}N_{out} + 159.87$	0.9901
eprop	$56.82N_{rec}N_{in} + 5.59N_{rec}N_{out} + 57.40N_{rec}N_{rec} + 10499.00$	0.9947
weight_update	$131.15N_{rec}N_{in} + 205.02N_{rec}N_{out} + 114.77N_{rec}N_{rec} + 7055.50$	0.9954
Total_no_update	$86.22N_{rec}N_{in} + 38.05N_{rec}N_{out} + 79.24N_{rec}N_{rec} + 86713.00$	0.994

1.3 Compare one memory with two memory

To find out how larger our model can be to fit in one PE and compare it with one PE that uses two PE's memory, we used formulas in Tables S1 and S2 and plotted the Figure S1. In these figure the number of input neurons is 80 and the number of output neurons is 12 and we changed the number of recurrent neurons from zero to 80.

In one PE, we are limited to 68KB of data memory. But if we use another memory, we can add 128KB of slow SRAM. So in one PE with two memories we have access to 196KB SRAM. If we add another slow memory, a PE has access to 324KB SRAM.

Figure S1 B shows that in one PE we can implement 21 recurrent neurons, for one PE with two memories we are limited to 50 recurrent neurons and for a PE with three memory the limitation is 77 recurrent neurons. For implementing our model with 120 recurrent neurons, one PE is not enough. We should parallelize our algorithm to use multi-PE and run the algorithm parallel on different PEs synchronously.

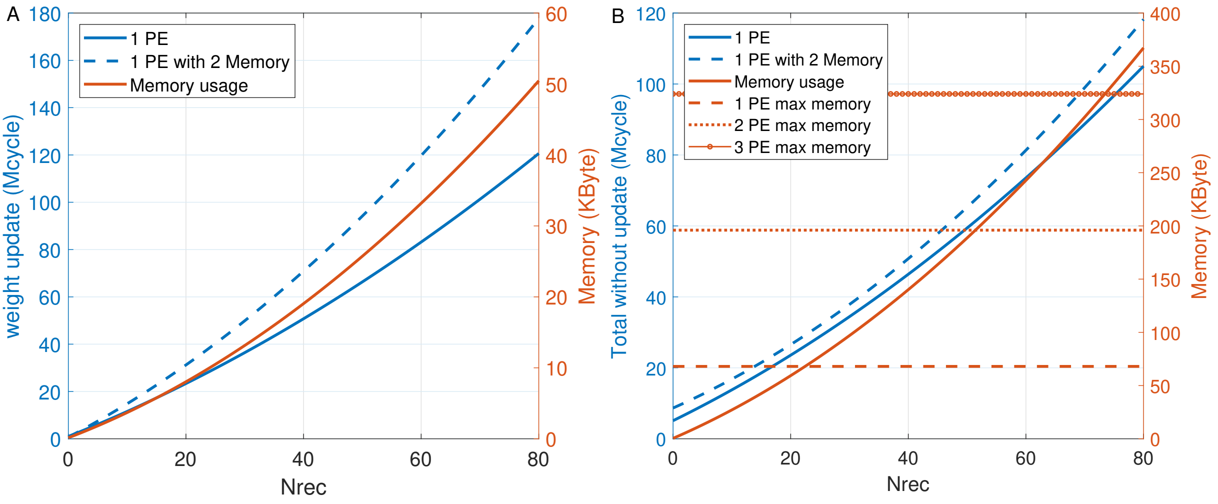

Figure S1. Compare clock cycle and memory usage for one PE with one and two memory. Clock cycle is for 1 mini batch and is predicted by fitted function in Tables S1 and S2 and showed in blue lines. Memory is calculated by using equation for e-prop in the main paper and it is plotted in red lines. The horizontal axis is number of recurrent neurons. (A) The clock cycle and memory for weight_update function is shown. (B) The clock cycle and memory for all function except weight_update function is shown. Also the maximum available memory if one uses one, two or three memory(s) are plotted by red dash lines

2 MEMORY COMPUTATION FOR (ZHANG ET AL., 2018) PAPER

In (Zhang et al., 2018) paper, first they trained the model by using 32-bit floating point data format on GPU for different architectures such as depthwise separable CNN (DS-CNN), CNN and GRU. Then they quantized the deep neural network (DNN, a standard feed-forward neural network) model and applied inference on ARM Cortex-M7 microcontroller which occupied about 70KB memory. For a fair comparison we consider memory usage for training with 32-bit floating point data format. It needs 7.84KB (=49x40x4Byte) to store input data. First layer is a convolution layer with 64 features and stride of 2x2. The output of first layer would be 125KB (=64x49x40/4x4Byte). Then there are 4 layers of depthwise separable CNN with 64 feature and stride of 1x1, which means each layer needs another 125KB for intermediate result. So the memory for storing intermediate variable of all layer would be 250KB. According to the paper the memory required for saving the 8-bit weights is 38.6KB, which for 32-bit weights it become 154KB. In back propagation path, we need to store the gradients of weights and also at least 125KB (=64x49x40/4x4Byte) for intermediate variables storage, so it becomes 434KB (=2x154.4+125.44) for weights, gradients and intermediate values in back path. Finally we need at least 692KB memory for training a 32-bit floating point model.

3 POWER MEASUREMENT

In Figure S2 the power measurement for GPU is plotted. The sampling rate period is 30 second. It takes 1 hour and 58 minutes to train e-prop. During this time the average power usage is 91.3W. Also a CPU and a 100GB RAM are connected to the GPU. It takes 6 minutes to read the whole preprocess dataset and copy it into the RAM. During training more than 40GB of RAM was used. The CPU and RAM power usage is not considered here.

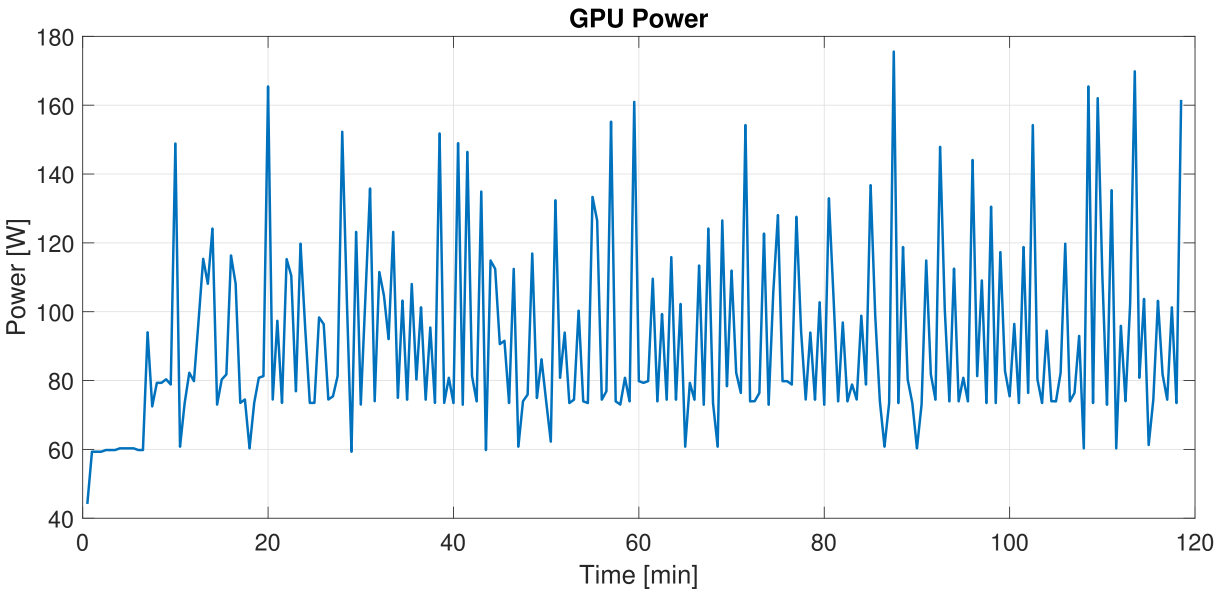

Figure S2. GPU power measurement during e-prop training.
